# New Residence-Based Formula for Ryan White HIV/AIDS Program Funding: Projected Jurisdictional Impacts, 2025–2030

**DOI:** 10.1093/ofid/ofag621

**Published:** 2026-10-03

**Authors:** Reanna K Panagides, Erin Q Rogers, Amy Killelea, Tim Horn, Jessica Keim-Malpass, Stephen D Turner, Amber Steen, Kathleen A McManus

**Affiliations:** Division of Infectious Diseases & International Health, University of Virginia School of Medicine, Charlottesville, Virginia, USA; Division of Infectious Diseases & International Health, University of Virginia School of Medicine, Charlottesville, Virginia, USA; McCourt School of Public Policy, Georgetown University, Washington, District of Columbia, USA; NASTAD, Washington, District of Columbia, USA; School of Nursing, University of Virginia, Charlottesville, Virginia, USA; School of Data Science, University of Virginia, Charlottesville, Virginia, USA; School of Nursing, University of Virginia, Charlottesville, Virginia, USA; Division of Infectious Diseases & International Health, University of Virginia School of Medicine, Charlottesville, Virginia, USA

**Keywords:** ending the HIV epidemic, medicaid expansion, resource allocation, health policy, Ryan White HIV/AIDS Program

## Abstract

Ryan White HIV/AIDS Part A/B formula awards were historically based on residence at HIV (HIV-1) diagnosis. We modeled the recent switch to current-residence formulas, calculating percent change in awards from 2025 to 2030. Combined awards rise overall, but results vary by region, Medicaid expansion status, and Ending the HIV Epidemic designation.

Ryan White HIV/AIDS (RWHAP) is the federal payer of last resort for HIV care for uninsured or underinsured low-income people with HIV (PWH). RWHAP Part A and Part B formula awards have historically been calculated using each person's residence at the time of HIV diagnosis. Starting in FY2026, the Health Resources and Services Administration (HRSA) will base funding on most recent known address instead of residence at time of HIV diagnosis, phasing in the change over 5 years through FY2030 [1]. Because PWH often move after diagnosis, basing funding residence at time of HIV diagnosis could be an inefficient way to target resources based on need; the new approach aligns RWHAP with other Department of Health and Human Services (HHS) formulas that allocate by current residence [1]. While the approach will allocate resources more accurately, award reductions may strain jurisdictions that rely on RWHAP to fill gaps not covered by broader health systems, potentially affecting staffing, services, and medication assistance that sustain viral suppression.

Prior research has documented geographic and policy-related disparities in HIV care access and outcomes [2, 3]; however, the distribution of projected formula changes across these contexts has not been described. These concerns may be especially relevant in Medicaid nonexpansion states, where PWH are less likely to have Medicaid coverage [4], Ending the HIV Epidemic (EHE) jurisdictions, which by definition have more HIV diagnoses [5], and the Southern United States, which experiences a disproportionate percentage of new HIV diagnoses and HIV-related deaths each year [6] and has fewer Medicaid expansion states [7]. We identified funding changes across RWHAP Part A, Part B, and combined awards for these jurisdiction types.

## METHODS

Projected RWHAP Part A and Part B awards for FY2025–FY2030 were obtained from the HRSA notice (90 FR 50747; November 10, 2025), announcing phased implementation of a revised approach to the RWHAP funding formula using most recent known address instead of residence at HIV diagnosis. We then classified jurisdictions by FY2025 Medicaid expansion status (KFF, 2025) [7], EHE designation (HHS, 2026) [5], and US Census region (2023) [8]. We categorized Part A recipients as EHE if their service area included at least 1 of the 48 HHS EHE-designated counties, Washington DC, or San Juan, Puerto Rico (n = 39). Part B and combined state/territory grantees were classified based on the 7 states designated as EHE priority jurisdictions for their substantial rural HIV burden [5]. Each Part A award was attributed in full to the state of its HRSA-designated recipient jurisdiction, even where the grant area spanned multiple states; combined awards were the sum of state-attributed Part A and Part B awards. We calculated FY2025–FY2030 change for Part A, Part B, and combined awards, overall and stratified by these factors. DC was categorized as part of the South. Consistent with RWHAP annual reports, we included 3 territories (Puerto Rico, Guam, US Virgin Islands) [9] in overall and regional analyses only. Jurisdictions with the highest 5-year percent change were highlighted. Analyses used R, version 4.3.1.

## RESULTS

Combined RWHAP Part A and B funding is projected to increase modestly by FY2030 because of the formula shift, but the average masks wide variation across jurisdictions, offsetting Part A and Part B trends. Figure 1 illustrates the geographic distribution of 5-year percent changes, with state/territory-level detail in Supplementary Table 1 and Figure 1A–C. Overall, jurisdictions are projected to experience a mean (SD) 3.0% (8.9%) increase in combined RWHAP Part A and B funding, a −0.3% (9.1%) change in Part A, and a 3.1% (9.0%) increase in Part B by FY2030 (Table 1). Changes vary widely: for combined funding and Part B, 2 jurisdictions decline and 9 increase by >10%; for Part A, 7 decline and 7 increase by >10% (Supplementary Table 2).

**Figure 1.**
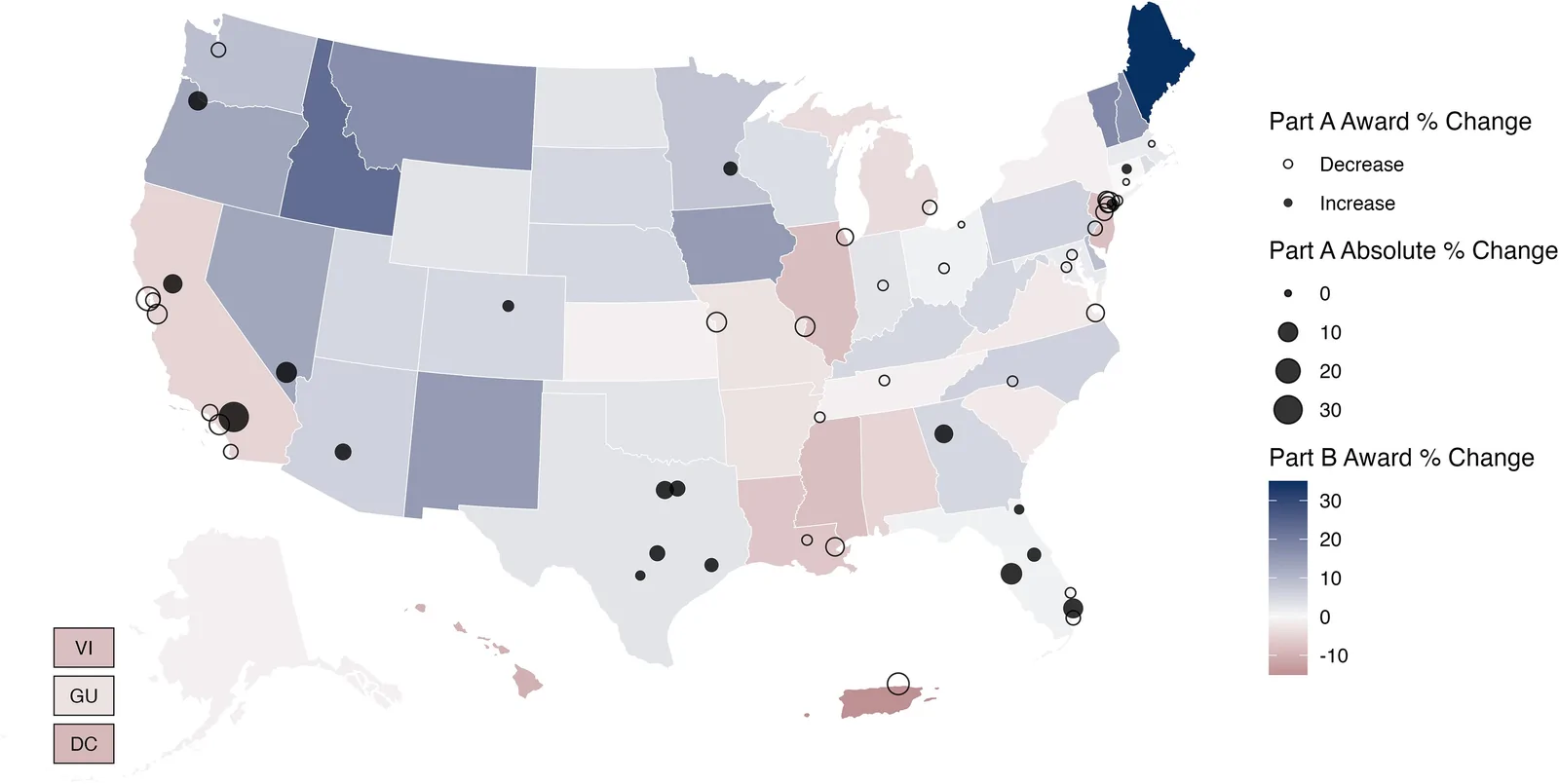
Estimated 5-year change (%) in RWHAP funding across Part A and Part B grantees, 2025–2030. Figure 1 illustrates the 5-year percent change in RWHAP Part A and B funding across jurisdictions from 2025 to 2030. For jurisdictions receiving Part B funding, the color and shade indicate the direction and magnitude of funding change. The color scale is centered on white (no change), with blue representing an increase and red representing a decrease in funding. The shade intensity reflects the absolute change in funding. For each city receiving Part A funding, a filled or empty circle indicates an increase or decrease, respectively, while the circle's size represents the absolute change in funding. Abbreviation: RWHAP, Ryan White HIV/AIDS Program.

**Table 1.** Average Estimated Five-Year Percent Change in RWHAP Part A and B Formula Awards, 2025–2030

	Part A n = 52				Part B n = 54				Combined Part A + Part B^a^ n = 54			
Category	No. (%)	2025, Mean (SD), $	2030, Mean (SD), $	Average 5-y % Change ^b^	No. (%)	2025, Mean (SD), $	2030, Mean (SD), $	Average 5-y % Change ^b^	No. (%)	2025, Mean (SD), $	2030, Mean (SD), $	Average 5-y % Change ^b^
Overall	52	12 451 853 (14 231 758)	12 451 853 (14 405 009)	−0.3 (9.1)	54	21 849 480 (32 334 741)	21 845 141 (32 106 305)	3.1 (9.0)	54	33 840 154 (54 910 203)	33 835 814 (54 814 525)	3.0 (8.9)
Medicaid expansion status	…	…	…	…	…	…	…	…	…	…	…	…
Adopted	37 (72.5%)	12 143 159 (15 903 841)	11 939 307 (15 962 862)	−1.7 (9.5)	41 (80.4)	18 603 166 (28 540 942)	18 531 042 (27 617 199)	4.8 (9.3)	41 (80.4)	29 561 626 (50 112 322)	29 305 539 (48 825 850)	4.5 (9.1)
Not adopted^c^	14 (27.5%)	13 417 061 (9 620 453)	14 068 502 (10 189 290)	4.4 (5.6)	10 (19.6)	39 512 100 (45 185 722)	40 102 008 (46 451 367)	−0.1 (4.1)	10 (19.6)	58 295 985 (74 837 964)	59 797 910 (77 487 635)	0.2 (4.4)
EHE-designated	…	…	…	…	…	…	…	…	…	…	…	…
No	13 (25%)	4 844 940 (1 405 564)	4 707 811 (1 438 233)	−3 (6.8)	44 (86.3)	23 992 704 (35 346 079)	24 112 764 (35 068 709)	4.7 (8.9)	44 (86.3)	38 225 294 (59 893 175)	38 408 821 (59 743 709)	4.6 (8.7)
Yes^d^	39 (75%)	14 987 491 (15 646 579)	15 033 201 (15 826 703)	0.5 (9.7)	7 (13.7)	14 595 974 (6 036 983)	14 261 595 (5 781 547)	−1.9 (4.5)	7 (13.7)	16 153 368 (7 101 241)	15 645 436 (6 615 346)	−2.3 (5)
Region	…	…	…	…	…	…	…	…	…	…	…	…
Midwest	8 (15.4%)	8 682 747 (7 878 838)	8 261 486 (7 281 172)	−3.9 (5.4)	12 (22.2)	12 220 370 (12 450 963)	12 020 615 (11 549 483)	2.3 (6.0)	12 (22.2)	18 008 868 (20 417 521)	17 528 272 (18 953 488)	1.7 (6.3)
Northeast	10 (19.2%)	16 528 101 (26 503 221)	16 456 925 (27 154 007)	−3.4 (4.5)	9 (16.7)	26 414 001 (38 942 466)	26 421 758 (38 603 222)	8.0 (13.0)	9 (16.7)	44 778 558 (69 326 036)	44 707 230 (69 465 299)	7.7 (13.0)
South^e^	20 (38.5%)	13 052 905 (9 617 183)	13 421 348 (10 054 276)	2.0 (6.3)	17 (31.5)	32 463 360 (35 476 275)	32 797 719 (36 515 761)	0.1 (5.3)	17 (31.5)	47 819 719 (58 376 174)	48 587 540 (60 466 019)	0.5 (5.0)
West	13 (25%)	10 871 896 (11 151 231)	10 740 407 (10 342 679)	1.7 (14.5)	13 (24.1)	17 046 401 (37 662 169)	17 014 491 (35 705 503)	7.2 (9.2)	13 (24.1)	27 918 297 (65 933 214)	27 754 898 (62 909 340)	6.7 (9.1)
US territory^f^	1 (1.9%)	10 360 633 (NA)^g^	8 782 983 (NA)^g^	−15.0 (NA)^g^	3 (5.6)	7 340 382 (11 638 663)	6 281 600 (9 877 144)	−8.7 (6.0)	3 (5.6)	10 793 926 (17 618 661)	9 209 261 (14 946 363)	−8.5 (6.1)

Abbreviations: EHE, Ending the HIV Epidemic; RWHAP, Ryan White HIV/AIDS Program.

^a^Includes the sum of Part A and B funding lines for US state and territory jurisdictions.

^b^Average 5-year % change is the mean of jurisdictions’ individual percent changes.

^c^States that have not adopted Medicaid expansion: Alabama, Florida, Georgia, Kansas, Mississippi, South Carolina, Tennessee, Texas, Wisconsin, Wyoming.

^d^States designated as EHE priority jurisdictions (applies to Part B and combined funding): Alabama, Arkansas, Kentucky, Mississippi, Missouri, Oklahoma, South Carolina. Part A jurisdictions were classified separately as EHE-designated based on 48 EHE priority counties (plus Washington DC and San Juan Municipio, PR).

^e^District of Columbia categorized in the Southern region of the United States.

^f^US territories include Puerto Rico, Guam, US Virgin Islands.

^g^San Juan, PR, was the only jurisdiction that receives Part A funding among US territories; therefore, standard deviation is not applicable.

Combined-funding gains concentrate in Medicaid expansion states (mean [SD], 4.5% [9.1%]) vs near-0 change in nonexpansion states (mean [SD], 0.2% [4.4%]), a gap driven largely by Part B. For Part A, the mean change (SD) is −1.7% (9.5%) in expansion compared with 4.4% (5.6%) in nonexpansion states; for Part B, 4.8% (9.3%) vs −0.1% (4.1%). Although nonexpansion averages near 0, some states will see notable decreases, including Mississippi (−8%) and Alabama (−5%), both Medicaid nonexpansion, EHE-designated states (Supplementary Figure 3A–C).

EHE designation shows divergent patterns by funding stream. Part A jurisdictions in an EHE-designated county will see a mean change (SD) of 0.5% (9.7%), compared with −3.0% (6.8%) in non-EHE-designated jurisdictions. For Part B and combined funding, EHE priority states will see larger losses than non-EHE-designated states: −1.9% (4.5%) vs 4.7% (8.9%) for Part B and −2.3% (5.0%) vs 4.6% (8.7%) for combined. Five of the 7 EHE-designated states will see Part B decreases, with Mississippi (−8%) and Alabama (−5%) projected to experience the largest reductions (Supplementary Figure 2A–C).

Regional trends are uneven, with the Northeast and the West projected to gain most and US territories to lose most. Mean 5-year changes in combined funding (SD) are 1.7% (6.3%) for the Midwest, 7.7% (13.0%) for the Northeast, 0.5% (5.0%) for the South, 6.7% (9.1%) for the West, and −8.5% (6.1%) for US territories. Northeastern and Western gains are driven by Part B increases; Southern changes are minimal and driven by Part A awards (Table 1).

## DISCUSSION

While the change to the RWHAP Part A and B funding formula to allocate funding by recent address instead of residence at HIV diagnosis will lead to a more accurate per-person resource allocation, jurisdictions will experience variable changes in RWHAP funding across Medicaid expansion status, EHE status, and geographic region. Paradoxically, Medicaid expansion states are projected to receive larger increases in combined Part A and B funding, while nonexpansion states will experience only marginal growth despite greater reliance on RWHAP to fill gaps in HIV care. The Northeastern and Western states will have large mean increases in RWHAP Part B funding to states, while the Northeast and Midwest will see decreases in RWHAP Part A funding to metropolitan areas. Jurisdictions that will have increases in funding will need to be proactive in planning to ensure efficient use of awards to meet the needs of PWH. Lastly, Southern states should expect marginal mean increases in RWHAP Part A funding and almost no change to RWHAP Part B funding.

Overall, states in the Southern United States, states with EHE rural area designation, and states without Medicaid expansion will experience on average either decreases or only marginal increases in RWHAP Part B funding from 2025 to 2030. One study demonstrated that people with diagnosed HIV are more geographically mobile than the general population, and following Medicaid expansion, move-in rates among PWH were higher in expansion states than in nonexpansion states [10]. Medicaid expansion has also been associated with increases in HIV diagnoses, awareness of status, and PrEP use, meaning expansion states may capture more diagnosed PWH in their current residence-based counts while nonexpansion states undercount [11]. In nonexpansion states, PWH rely more heavily on RWHAP support, including AIDS Drug Assistance Programs (ADAPs), because they are more likely to be uninsured [4]. Jurisdictions in the Southern United States and with EHE designations are already more likely to have higher HIV funding needs because of structural barriers to care, which are more pronounced in these areas [12]. Large decreases in Part B funding in Medicaid nonexpansion and EHE jurisdictions, and in Part A funding among non-EHE-designated jurisdictions, will necessitate jurisdictional planning to ensure continuity of core medical services and ancillary services, such as case management, housing, and transportation assistance, that mitigate these barriers and support PWH's viral suppression.

We highlight jurisdictions that will experience large shifts in funding essential to supporting PWH, and these findings require careful contextual interpretation accounting for broader structural influences impacting HIV care. Results from this report should be interpreted within an intersectional lens incorporating contextual factors such as ADAPs considering cost containment measures [13] and the subsequent implementation of some of these measures [14].

This analysis has limitations. Our projections are based on the award figures HRSA published for the beginning and end of the phase in FY2025–FY2030. We did not adjust these projections to account for potential changes in migration, changes in HIV prevalence, or demographic shifts over the period. Because Part A awards were attributed to the HRSA-designated recipient state, funds serving residents of adjacent states within multistate metropolitan areas are not captured. Subgroup standard deviations frequently exceed the corresponding means, so subgroup averages should be interpreted as central tendencies rather than uniform effects. Finally, the mobility and ascertainment mechanisms we described are inferred from related literature rather than modeled directly here.

While the transition to RWHAP funding based on last address rather than residence at HIV diagnosis provides a more accurate per-person allocation, our findings highlight divergent jurisdictional impacts. Funding is increasing in states that have already expanded health care access, while EHE-designated states and US territories will see decreases, potentially impacting care and viral suppression. Maintaining the integrity of the HIV care safety net during this transition will require proactive, context-specific planning. Future policy discussions should consider how these data-driven updates interact with existing structural variations in state-level health care access.

## Supplementary Material

ofag621_Supplementary_Data

## Supplementary Data

Supplementary materials are available at *Open Forum Infectious Diseases* online. Consisting of data provided by the authors to benefit the reader, the posted materials are not copyedited and are the sole responsibility of the authors, so questions or comments should be addressed to the corresponding author.
