# Supplementary material for: New Residence-Based Formula for Ryan White HIV/AIDS Program Funding: Projected Jurisdictional Impacts, 2025–2030
Source: Open Forum Infect Dis. 2026 Oct 3;13(10):ofag621. doi: 10.1093/ofid/ofag621 (PMC13678204; doi:10.1093/ofid/ofag621)
Supplement: Supplementary file 1 [file ofag621_supplementary_data.docx]

**New Residence-Based Formula for Ryan White HIV/AIDS Program Funding: Projected Jurisdictional Impacts, 2025-2030**

**Online Only Supplemental Material - Table of Contents**

eTable 1. Estimated 5-year Percent Change RWHAP Part A, B, and Combined Awards by Jurisdiction, 2025-2030…………………………………………………………………..2

eTable 2. Direction and Magnitude of 5-year % Change RWHAP Part A and B Formula Awards, 2025-2030…………………………………………………………………………..8

eFigure 1a. RWHAP Part A Grantees by percent change of Part A FY25-FY30, from largest to smallest………………………………………………………………………….....9

eFigure 1b. RWHAP Part B Grantees by percent change of Part B FY25-FY30, from largest to smallest…………………………………………………………………………….10

eFigure 1c. Jurisdictions by percent change of combined RWHAP Part A + Part B FY25-FY30, from largest to smallest……………………………………………………………….11

eFigure 2a. RWHAP Part A Grantees by percent change of Part A FY25-FY30, colored by EHE Designation………………………………………………………………………12

eFigure 2b. RWHAP Part B Grantees by percent change of Part B FY25-FY30, colored by EHE Designation…………………………………………………………………………13

eFigure 2c. Jurisdictions by percent change of combined RWHAP Part A + Part B FY25-FY30, colored by EHE Designation…………………………………………………14

eFigure 3a. RWHAP Part A Grantees by percent change of Part A FY25-FY30, colored by Medicaid Expansion Status………………………………………………………15

eFigure 3b. RWHAP Part B Grantees by percent change of Part B FY25-FY30, colored by Medicaid Expansion Status………………………………………………………16

eFigure 3c. Jurisdictions by percent change of combined RWHAP Part A + Part B FY25-FY30, colored by Medicaid Expansion Status…………………………17

**eTable 1. Estimated 5-year Percent Change RWHAP Part A, B, and Combined Awards by Jurisdiction, 2025-2030**

| **State** | **Grantee ^a^** | **FY2025** | **FY2030** | **5-year % Change** |
| --- | --- | --- | --- | --- |
| Alabama | Part B | $20,126,410 | $19,200,916 | -4.6 |
|  | Combined Part A and B | $20,126,410 | $19,200,916 | -4.6 |
| Alaska | Part B | $1,057,741 | $1,045,127 | -1.2 |
|  | Combined Part A and B | $1,057,741 | $1,045,127 | -1.2 |
| Arizona | Part B | $17,593,880 | $18,637,862 | 5.9 |
|  | Phoenix | $11,049,463 | $11,774,426 | 6.6 |
|  | Combined Part A and B | $28,643,343 | $30,412,288 | 6.2 |
| Arkansas | Part B | $8,467,112 | $8,194,525 | -3.2 |
|  | Combined Part A and B | $8,467,112 | $8,194,525 | -3.2 |
| California | Part B | $140,896,380 | $134,016,569 | -4.9 |
|  | Los Angeles | $46,295,740 | $43,464,912 | -6.1 |
|  | Oakland | $7,360,883 | $7,078,648 | -3.8 |
|  | Sacramento | $3,832,752 | $4,264,397 | 11.3 |
|  | San Bernardino | $9,283,666 | $12,720,488 | 37.0 |
|  | San Diego | $12,325,778 | $11,805,331 | -4.2 |
|  | San Francisco | $14,841,000 | $11,972,966 | -19.3 |
|  | San Jose | $3,496,090 | $3,110,454 | -11.0 |
|  | Santa Ana | $6,679,132 | $5,902,938 | -11.6 |
|  | Combined Part A and B | $245,011,421 | $234,336,703 | -4.4 |
| Colorado | Part B | $13,159,341 | $13,870,517 | 5.4 |
|  | Denver | $7,340,580 | $7,481,041 | 1.9 |
|  | Combined Part A and B | $20,499,921 | $21,351,558 | 4.2 |
| Connecticut | Part B | $10,028,100 | $9,980,203 | -0.5 |
|  | Hartford | $2,944,998 | $2,975,179 | 1.0 |
|  | New Haven | $5,273,601 | $5,282,556 | 0.2 |
|  | Combined Part A and B | $18,246,699 | $18,237,938 | -0.1 |
| **State** | **Grantee ^a^** | **FY2025** | **FY2030** | **5-year % Change** |
| Delaware | Part B | $4,559,579 | $4,978,872 | 9.2 |
|  | Combined Part A and B | $4,559,579 | $4,978,872 | 9.2 |
| **State** | **Grantee ^a^** | **FY2025** | **FY2030** | **5-year % Change** |
| District of Columbia | Part B | $15,877,251 | $14,378,464 | -9.4 |
|  | Washington | $32,044,482 | $31,800,861 | -0.8 |
|  | Combined Part A and B | $47,921,733 | $46,179,325 | -3.6 |
| Florida | Part B | $117,953,889 | $119,674,395 | 1.5 |
|  | Fort Lauderdale | $16,023,456 | $17,871,267 | 11.5 |
|  | Jacksonville | $6,108,505 | $6,154,971 | 0.8 |
|  | Miami | $26,788,349 | $25,681,190 | -4.1 |
|  | Orlando | $10,939,376 | $11,406,483 | 4.3 |
|  | Tampa | $10,650,266 | $12,207,013 | 14.6 |
|  | West Palm Beach | $7,428,827 | $7,382,922 | -0.6 |
|  | Combined Part A and B | $195,892,668 | $200,378,241 | 2.3 |
| Georgia | Part B | $65,937,492 | $69,375,111 | 5.2 |
|  | Atlanta | $32,339,493 | $35,514,205 | 9.8 |
|  | Combined Part A and B | $98,276,985 | $104,889,316 | 6.7 |
| Guam | Part B | $280,781 | $272,959 | -2.8 |
|  | Combined Part A and B | $280,781 | $272,959 | -2.8 |
| Hawaii | Part B | $3,352,217 | $3,017,570 | -10.0 |
|  | Combined Part A and B | $3,352,217 | $3,017,570 | -10.0 |
| Idaho | Part B | $1,567,448 | $1,929,082 | 23.1 |
|  | Combined Part A and B | $1,567,448 | $1,929,082 | 23.1 |
| Illinois | Part B | $43,632,706 | $39,940,798 | -8.5 |
|  | Chicago | $27,666,573 | $25,756,452 | -6.9 |
|  | Combined Part A and B | $71,299,279 | $65,697,250 | -7.9 |
| Indiana | Part B | $14,826,971 | $15,309,391 | 3.2 |
|  | Indianapolis | $4,910,030 | $4,850,989 | -1.2 |
|  | Combined Part A and B | $19,737,001 | $20,160,380 | 2.1 |
| Iowa | Part B | $3,671,105 | $4,232,858 | 15.3 |
|  | Combined Part A and B | $3,671,105 | $4,232,858 | 15.3 |
| Kansas | Part B | $3,874,733 | $3,825,365 | -1.3 |
|  | Combined Part A and B | $3,874,733 | $3,825,365 | -1.3 |
| **State** | **Grantee ^a^** | **FY2025** | **FY2030** | **5-year % Change** |
| Kentucky | Part B | $10,928,564 | $11,522,927 | 5.4 |
|  | Combined Part A and B | $10,928,564 | $11,522,927 | 5.4 |
| Louisiana | Part B | $23,907,778 | $22,249,514 | -6.9 |
|  | Baton Rouge | $4,551,433 | $4,498,838 | -1.2 |
|  | New Orleans | $8,058,405 | $7,323,525 | -9.1 |
|  | Combined Part A and B | $36,517,616 | $34,071,877 | -6.7 |
| Maine | Part B | $1,744,812 | $2,354,230 | 34.9 |
|  | Combined Part A and B | $1,744,812 | $2,354,230 | 34.9 |
| Maryland | Part B | $31,501,279 | $31,977,961 | 1.5 |
|  | Baltimore | $15,660,695 | $15,512,462 | -0.9 |
|  | Combined Part A and B | $47,161,974 | $47,490,423 | 0.7 |
| Massachusetts | Part B | $20,049,557 | $20,359,769 | 1.6 |
|  | Boston | $15,183,838 | $15,240,481 | 0.4 |
|  | Combined Part A and B | $35,233,395 | $35,600,250 | 1.0 |
| Michigan | Part B | $18,646,797 | $17,884,755 | -4.1 |
|  | Detroit | $10,075,549 | $9,688,708 | -3.8 |
|  | Combined Part A and B | $28,722,346 | $27,573,463 | -4.0 |
| Minnesota | Part B | $8,739,953 | $9,396,852 | 7.5 |
|  | Minneapolis | $6,129,008 | $6,384,904 | 4.2 |
|  | Combined Part A and B | $14,868,961 | $15,781,756 | 6.1 |
| Mississippi | Part B | $14,270,758 | $13,102,333 | -8.2 |
|  | Combined Part A and B | $14,270,758 | $13,102,333 | -8.2 |
| Missouri | Part B | $13,771,559 | $13,311,260 | -3.3 |
|  | Kansas City ^a^ | $4,513,410 | $4,021,761 | -10.9 |
|  | Saint Louis | $6,388,344 | $5,665,127 | -11.3 |
|  | Combined Part A and B | $24,673,313 | $22,998,148 | -6.8 |
| Montana | Part B | $868,081 | $1,013,465 | 16.8 |
|  | Combined Part A and B | $868,081 | $1,013,465 | 16.8 |
| Nebraska | Part B | $3,366,792 | $3,533,192 | 4.9 |
|  | Combined Part A and B | $3,366,792 | $3,533,192 | 4.9 |
| Nevada | Part B | $10,233,918 | $11,537,634 | 12.7 |
| **State** | **Grantee ^a^** | **FY2025** | **FY2030** | **5-year % Change** |
|  | Las Vegas | $7,551,492 | $8,572,451 | 13.5 |
|  | Combined Part A and B | $17,785,410 | $20,110,085 | 13.1 |
| New Hampshire | Part B | $1,408,963 | $1,631,599 | 15.8 |
|  | Combined Part A and B | $1,408,963 | $1,631,599 | 15.8 |
| New Jersey | Part B | $36,785,442 | $33,986,480 | -7.6 |
|  | New Brunswick | $2,803,890 | $2,607,538 | -7.0 |
|  | Newark | $12,277,381 | $11,500,568 | -6.3 |
|  | Paterson | $3,918,044 | $3,648,121 | -6.9 |
|  | Secaucus | $4,797,408 | $4,207,547 | -12.3 |
|  | Combined Part A and B | $60,582,165 | $55,950,254 | -7.7 |
| New Mexico | Part B | $4,461,845 | $5,120,231 | 14.8 |
|  | Combined Part A and B | $4,461,845 | $5,120,231 | 14.8 |
| New York | Part B | $121,693,828 | $120,738,999 | -0.8 |
|  | Mineola | $5,653,528 | $5,579,521 | -1.3 |
|  | New York | $89,662,231 | $91,619,685 | 2.2 |
|  | Combined Part A and B | $217,009,587 | $217,938,205 | 0.4 |
| North Carolina | Part B | $36,933,968 | $38,986,095 | 5.6 |
|  | Charlotte | $6,913,452 | $6,831,651 | -1.2 |
|  | Combined Part A and B | $43,847,420 | $45,817,746 | 4.5 |
| North Dakota | Part B | $865,271 | $890,261 | 2.9 |
|  | Combined Part A and B | $865,271 | $890,261 | 2.9 |
| Ohio | Part B | $25,253,858 | $25,546,610 | 1.2 |
|  | Cleveland | $4,772,432 | $4,776,087 | 0.1 |
|  | Columbus | $5,006,629 | $4,947,863 | -1.2 |
|  | Combined Part A and B | $35,032,919 | $35,270,560 | 0.7 |
| Oklahoma | Part B | $9,505,525 | $9,774,719 | 2.8 |
|  | Combined Part A and B | $9,505,525 | $9,774,719 | 2.8 |
| Oregon | Part B | $6,647,795 | $7,528,164 | 13.2 |
|  | Portland | $4,088,295 | $4,557,107 | 11.5 |
|  | Combined Part A and B | $10,736,090 | $12,085,271 | 12.6 |
| Pennsylvania | Part B | $41,565,377 | $43,980,468 | 5.8 |
| **State** | **Grantee ^a^** | **FY2025** | **FY2030** | **5-year % Change** |
|  | Philadelphia | $22,766,093 | $21,908,052 | -3.8 |
|  | Combined Part A and B | $64,331,470 | $65,888,520 | 2.4 |
| Puerto Rico | Part B | $20,773,717 | $17,681,166 | -14.9 |
|  | San Juan | $10,360,633 | $8,782,983 | -15.2 |
|  | Combined Part A and B | $31,134,350 | $26,464,149 | -15.0 |
| Rhode Island | Part B | $3,586,062 | $3,743,729 | 4.4 |
|  | Combined Part A and B | $3,586,062 | $3,743,729 | 4.4 |
| South Carolina | Part B | $25,101,893 | $24,724,486 | -1.5 |
|  | Combined Part A and B | $25,101,893 | $24,724,486 | -1.5 |
| South Dakota | Part B | $1,018,404 | $1,067,840 | 4.8 |
|  | Combined Part A and B | $1,018,404 | $1,067,840 | 4.8 |
| Tennessee | Part B | $19,798,995 | $19,681,823 | -0.6 |
|  | Memphis | $6,902,702 | $6,819,821 | -1.2 |
|  | Nashville | $4,443,640 | $4,387,638 | -1.3 |
|  | Combined Part A and B | $31,145,337 | $30,889,282 | -0.8 |
| Texas | Part B | $118,321,330 | $121,348,695 | 2.6 |
|  | Austin | $5,702,606 | $6,035,474 | 5.8 |
|  | Dallas | $21,184,061 | $22,477,465 | 6.1 |
|  | Fort Worth | $5,547,578 | $6,022,318 | 8.6 |
|  | Houston | $27,558,403 | $28,741,745 | 4.3 |
|  | San Antonio | $6,221,586 | $6,256,515 | 0.6 |
|  | Combined Part A and B | $184,535,564 | $190,882,212 | 3.4 |
| Utah | Part B | $5,084,544 | $5,315,691 | 4.5 |
|  | Combined Part A and B | $5,084,544 | $5,315,691 | 4.5 |
| Vermont | Part B | $863,866 | $1,020,348 | 18.1 |
|  | Combined Part A and B | $863,866 | $1,020,348 | 18.1 |
| Virgin Islands | Part B | $966,648 | $890,676 | -7.9 |
|  | Combined Part A and B | $966,648 | $890,676 | -7.9 |
| Virginia | Part B | $25,905,308 | $25,469,040 | -1.7 |
|  | Norfolk | $5,990,787 | $5,500,600 | -8.2 |
|  | Combined Part A and B | $31,896,095 | $30,969,640 | -2.9 |
| **State** | **Grantee ^a^** | **FY2025** | **FY2030** | **5-year % Change** |
| Washington | Part B | $15,920,820 | $17,377,714 | 9.2 |
|  | Seattle | $7,189,780 | $6,920,127 | -3.8 |
|  | Combined Part A and B | $23,110,600 | $24,297,841 | 5.1 |
| West Virginia | Part B | $2,779,989 | $2,921,342 | 5.1 |
|  | Combined Part A and B | $2,779,989 | $2,921,342 | 5.1 |
| Wisconsin | Part B | $8,976,297 | $9,308,193 | 3.7 |
|  | Combined Part A and B | $8,976,297 | $9,308,193 | 3.7 |
| Wyoming | Part B | $759,202 | $778,758 | 2.6 |
|  | Combined Part A and B | $759,202 | $778,758 | 2.6 |

Abbreviation: RWHAP, Ryan White HIV/AIDS Program

^a^ Part A funding is allocated to major metropolitan areas. In some cases, these metropolitan areas may cross state lines. The Part A grantees were assigned to the city based on the classification included in the HRSA notice.

Note. Includes 50 states, the District of Columbia, Puerto Rico, Guam, and the U.S. Virgin Islands.

|  |  | **Part A**  **n = 52** | **Part B**  **n = 54** | **Combined**  **(Part A + Part B)** ^a^  **n = 54** |
| --- | --- | --- | --- | --- |
| **Direction** | **Magnitude of Change** | **n (%)** | **n (%)** | **n (%)** |
| Decrease | <5% | 16 (31%) | 10 (19%) | 10 (19%) |
|  | 5-10% | 7 (13%) | 8 (15%) | 7 (13%) |
|  | 10-15% | 5 (10%) | 1 (2%) | 1 (2%) |
|  | ≥15% | 2 (4%) | 1 (2%) | 1 (2%) |
| No Change | 0 % | 3 (6%) | 1 (2%) | 2 (4%) |
| Increase | <5% | 8 (15%) | 11 (20%) | 14 (26%) |
|  | 5-10% | 4 (8%) | 13 (24%) | 10 (19%) |
|  | 10-15% | 5 (10%) | 2 (4%) | 2 (4%) |
|  | ≥15% | 2 (4%) | 7 (13%) | 7 (13%) |

**eTable 2. Direction and Magnitude of 5-year % Change RWHAP Part A and B Formula Awards, 2025-2030**

Note. "No Change" indicates no change (0%). "<5%" represents changes less than 5% in either direction. "5-10%" represent changes of 5 to 9%, "10-15%" represent changes of 10 to 14% and "≥15%" represent changes of 15% and greater.

^a^ Includes the sum of Part A and B funding lines for U.S. State and territory jurisdictions.

**eFigure 1a. RWHAP Part A Grantees by percent change of Part A FY25-FY30, from largest to smallest**

 Note. eFigure 1a illustrates increases and decreases in funding allocation across jurisdictions receiving Part A funding from 2025 to 2030. Warm hues (red, orange yellow) indicate decreases to funding. Cool hues (blue) indicated increases to funding. Grey indicates a less than 5% change in either direction. The length of the horizontal bars corresponds with the absolute change in funding amount. "5-10%" represent changes of 5 to 9%, "10-15%" represent changes of 10 to 14% and "≥15%" represent changes of 15% and greater.

**eFigure 1b. RWHAP Part B Grantees by percent change of Part B FY25-FY30, from largest to smallest**

Note. eFigure 1b illustrates increases and decreases in funding allocation across jurisdictions receiving Part B funding from 2025 to 2030. Warm hues (red, orange yellow) indicate decreases to funding. Cool hues (blue) indicated increases to funding. Grey indicates a less than 5% change in either direction. The length of the horizontal bars corresponds with the absolute change in funding amount. "5-10%" represent changes of 5 to 9%, "10-15%" represent changes of 10 to 14% and "≥15%" represent changes of 15% and greater.

**eFigure 1c. Jurisdictions by percent change of combined RWHAP Part A + Part B FY25-FY30, from largest to smallest**

Note. eFigure 1c illustrates increases and decreases in funding allocation across jurisdictions receiving combined funding lines from 2025 to 2030. Warm hues (red, orange yellow) indicate decreases to funding. Cool hues (blue) indicated increases to funding. Grey indicates a less than 5% change in either direction. The length of the horizontal bars corresponds with the absolute change in funding amount. "5-10%" represent changes of 5 to 9%, "10-15%" represent changes of 10 to 14% and "≥15%" represent changes of 15% and greater.

**eFigure 2a. RWHAP Part A Grantees by percent change of Part A FY25-FY30, colored by EHE Designation**

Note. eFigure 2a illustrates the 5-year percent change in RWHAP Part A funding across jurisdictions, ordered from largest decrease to largest increase. Bars are colored by whether the jurisdiction’s county is designated as an Ending the HIV Epidemic (EHE) priority jurisdiction.

**eFigure 2b. RWHAP Part B Grantees by percent change of Part B FY25-FY30, colored by EHE Designation**

Note. eFigure 2b illustrates the 5-year percent change in RWHAP Part B funding across jurisdictions, ordered from largest decrease to largest increase. Bars are colored by whether the jurisdiction is designated as an Ending the HIV Epidemic (EHE) priority jurisdiction.

**eFigure 2c. Jurisdictions by percent change of combined RWHAP Part A + Part B FY25-FY30, colored by EHE Designation**

Note. eFigure 2c illustrates the 5-year percent change in combined RWHAP Part A and Part B funding across jurisdictions, ordered from largest decrease to largest increase. Bars are colored by whether the jurisdiction is designated as an Ending the HIV Epidemic (EHE) priority jurisdiction.

**eFigure 3a. RWHAP Part A Grantees by percent change of Part A FY25-FY30, colored by Medicaid Expansion Status**

Note. eFigure 3a illustrates the 5-year percent change in RWHAP Part A funding across jurisdictions, ordered from largest decrease to largest increase. Bars are colored by the state’s Medicaid expansion status as of 2025.

**eFigure 3b. RWHAP Part B Grantees by percent change of Part B FY25-FY30, colored by Medicaid Expansion Status**

Note. eFigure 3b illustrates the 5-year percent change in RWHAP Part B funding across jurisdictions, ordered from largest decrease to largest increase. Bars are colored by the jurisdiction’s Medicaid expansion status as of 2025.

**eFigure 3c. Jurisdictions by percent change of combined RWHAP Part A + Part B FY25-FY30, colored by Medicaid Expansion Status**

Note. eFigure 3c illustrates the 5-year percent change in combined RWHAP Part A and Part B funding across jurisdictions, ordered from largest decrease to largest increase. Bars are colored by the jurisdiction’s Medicaid expansion status as of 2025.
